# A Diet Diverse in Bamboo Parts is Important for Giant Panda (*Ailuropoda melanoleuca*) Metabolism and Health

**DOI:** 10.1038/s41598-017-03216-8

**Published:** 2017-06-13

**Authors:** Hairui Wang, Heju Zhong, Rong Hou, James Ayala, Guangmang Liu, Shibin Yuan, Zheng Yan, Wenping Zhang, Yuliang Liu, Kailai Cai, Zhigang Cai, He Huang, Zhihe Zhang, De Wu

**Affiliations:** 1 Key Laboratory for Animal Disease Resistance Nutrition of the Ministry of Education, Animal Nutrition Institute, Sichuan Agricultural University, Ya’an, 625014 China; 2 Chengdu Research Base of Giant Panda Breeding, Sichuan Key Laboratory of Conservation Biology for Endangered Wildlife, Chengdu, 610081 China; 3 Key Laboratory of Southwest China Wildlife Resources Conservation (China West Normal University), Ministry of Education, Nanchong, 637009 China

## Abstract

The aim of this study was to determine the metabolic response in giant pandas (*Ailuropoda melanoleuca*) to the consumption of certain parts of bamboo above ground growth. Giant pandas were provisioned with three species of bamboo: *Phyllostachys bissetii*, of which they only consume the culm (culm group); *Bashania fargesii*, of which they only consume the leaves (leaf group); and *Qiongzhuea opienensis*, of which they only consume the shoots (shoot group). The “culm” group absorbed the highest amount of calories and fiber, but was in short energy supply (depressed tricarboxylic acid cycle activity), and high fiber level diet might reduce the digestibility of protein. The “culm” and “leaf” groups absorbed less protein, and had a lower rate of body mass growth than the “shoot” group. Digestion of fiber requires energy input and yields low caloric extraction from the culm and leaf, and protein intake is important for increasing body mass. However, long-term consumption of shoots may have a potentially negative effect on the health because of high protein composition. Therefore, a balanced diet consisting of diverse plant parts of bamboo is important for the overall metabolic function and health of captive giant pandas.

## Introduction

The exclusive bamboo diet of giant pandas (*Ailuropoda melanoleuca*) is unique within the Order Carnivora and well documented in the literature. There are over 1,200 species of bamboo, of which giant pandas consume more than 60^1, 2^. The growth of the bamboo follows a cyclic temporal pattern, which results in seasonal nutritional changes within the different plant parts^3^. Pandas primarily eat shoots when shoots are available which is only for a few months of the year during the shooting season^3^. During the shooting season bamboo invests its energy into shoot growth, not leaf development, but at the end of the shooting season bamboo leaves increase in nutrient value as the plants put growth and energy into photosynthesis^4^. When the shooting season is over, pandas eat the bamboo leaves. In the winter the leaves lose much of their available nutrient value while the bamboo goes dormant. During this time the culms have highest available nutrient composition (compared to leaves, and when no shoots are available), so pandas eat culms. Because of the cyclicity of the bamboo growth, the giant pandas consume different parts accordingly.

In the wild, more than one kind of bamboo species is available for pandas for the most time of a year. Due to such factors as season, transportation, weather, etc., captive giant pandas are generally provided with only one species of bamboo. Thus, pandas in captivity primarily eat shoots in spring, leaves in summer and fall, and culms in winter and early spring before the shoots grow in the next cycle^5^. Adult giant pandas are provided with several kinds of bamboo species each year^6–8^. At the Chengdu Research Base of Giant Panda Breeding (Chengdu, Sichuan Province, China), three main species fed to pandas at this facility include: *Phyllostachys B*., of which pandas only consume the culms, *Bashania F*., of which they only consume the leaves, and the bamboo shoots of *Qiongzhuea O* ^9^. Such single-plant part provisioning may continue for several months. Previous studies show that the nutritional value varies greatly in different parts of the bamboo body^10–13^. Moreover, the bioavailability of different plant parts varies significantly^14, 15^. So, if the consumption of single-parts continues for a long period of time, it may potentially lead to nutritional imbalances and negatively impact the health of giant pandas.

The objectives of this study were to determine the affect of consuming only certain parts of bamboo on the metabolism and health of pandas through the use of metabolomics. Nuclear Magnetic Resonance Spectrometry (NMR), Gas Chromatography-Mass Spectrometry (GC-MS) and Liquid Chromatography-Mass Spectrometry (LC-MS) are currently widely adopted methods for metabolomics analysis and detection. For NMR sample preparation, the sample pre-treatment is simple or even unnecessary under certain conditions, thus it is non-destructive and will not affect the structure or property of the samples^16, 17^, however, NMR has low sensitivity. In comparison, both types of MS have feature high efficiency, great sensitivity, qualitative and quantitative determination, and can detect and identify multiple metabolites in a complex mixture. Because of these advantages, MS is extensively used in metabolomic analysis, however the technique has several disadvantages such as matrix interference and difficulty in data comparison^18^. Using both GC-MS and NMR metabolomic analysis, we examined the metabolites of the blood serum extracted from captive giant pandas which were fed diets of different composition. The nutrient digestibility, metabolomic data and biochemical indicators were collected for these animals. All of these parameters may provide a biological explanation for the metabolism and general health of pandas in response to consuming different plant parts of bamboo.

## Results

### Daily food intake, nutrient digestibility and body mass gain

The daily dry matter intake differed significantly when the pandas consumed different bamboo parts *ad libitum*. The level of daily dry matter intake, crude fiber and energy intake were higher (*p* < 0.05) in “culm” group than in “shoot” and “leaf” groups, while the amount of ether extract intake and crude protein intake were the lowest in “culm” group (*p* < 0.05). For the “shoot” group, the level of daily dry matter intake was the lowest (*p* < 0.05) among the three groups, while the crude protein intake, digestibility and amount digested were the highest among the three groups (*p* < 0.05). The “shoot” group also had lower level of crude fiber intake and energy intake in comparison with the “culm” (*p* < 0.05)and “leaf” groups. Compared with “culm” and “leaf” groups, “shoot” group had higher body mass gain (*p* < 0.05) (Table 1).

**Table 1:** Daily nutrient intake (dry matter basis), nutrient apparent digestibility and body mass gain of pandas.

Item	Treatments		
	Culm group	Leaf group	Shoot group
DM intake (g/d)	7088.45 ± 1467.97^a^	4030.59 ± 156.41^b^	2102.17 ± 483.82^c^
CP			
CP intake (g/d)	132.55 ± 37.46^b^	633.28 ± 43.80^a^	651.40 ± 153.31^a^
Digestible CP (g/d)	52.96 ± 16.23^c^	374.96 ± 44.15^b^	509.39 ± 77.40^a^
CP digestibility%	40.01 ± 11.59^c^	59.34 ± 7.28^b^	79.53 ± 8.53^a^
EE			
EE intake (g/d)	33.80 ± 7.10^c^	130.82 ± 12.34^a^	59.53 ± 13.77^b^
Digestible EE (g/d)	25.97 ± 5.47^b^	53.55 ± 16.11^a^	31.77 ± 9.90^ab^
EE digestibility%	77.08 ± 7.66^a^	41.43 ± 13.34^b^	57.84 ± 7.61^ab^
CF			
CF intake (g/d)	3225.41 ± 626.34^a^	978.95 ± 119.90^b^	357.78 ± 83.17^c^
Digestible CF (g/d)	1582.73 ± 162.97^a^	270.30 ± 157.91^b^	62.34 ± 12.86^b^
CF digestibility%	50.54 ± 10.67^a^	28.98 ± 19.03^ab^	18.11 ± 8.63^b^
Energy			
Energy intake (MJ/d)	134.14 ± 27.58^a^	75.43 ± 1.46^b^	37.00 ± 8.52^c^
Digestible energy (MJ/d)	69.37 ± 6.28^a^	35.28 ± 6.49^b^	14.80 ± 2.92^c^
Energy digestibility%	52.76 ± 8.82	46.88 ± 9.34	40.55 ± 5.29
Average daily gain (kg/d)	−0.07 ± 0.40^b^	0.06 ± 0.07^b^	0.23 ± 0.24^a^

Data represent the means ± SD. Different superscripts indicate significant difference (*p* < 0.05). The calculation of the CP, CF, EE and energy is based on the dry matter. Energy is only from the bamboo food. DM: dry matter; CP: crude protein; CF: crude fiber; EE: ether extract.

### Biochemical parameters

Clinical chemistry results show that BUN/CRE, BUN, TCHO, TG, VLDL, WBC and neutrophil levels were significantly different between these three groups (*p* < 0.05). Among these parameters, the BUN/CRE, BUN and WBC levels in the “shoot” group were much higher (*p* < 0.05) than in other two groups, and the Neutrophil% is higher in “shoot” group than in “leaf” (*p* < 0.05). And the TCHO, TG, VLDL levels in the “leaf” group were much higher (*p* < 0.05) than in the other two groups (Table 2).

**Table 2:** Conventional biochemical serum parameters from giant pandas kept on culm, leaf and shoot feeding regimes.

Parameter	Treatments		
	Culm group (n = 10)	Leaf group (n = 9)	Shoot group (n = 9)
ALP (U/L)	124.11 ± 24.96	114.33 ± 43.13	136.74 ± 26.36
BUN/CRE	0.02 ± 0.00^c^	0.06 ± 0.04^b^	0.11 ± 0.06^a^
BUN (mmol/L)	1.99 ± 0.67^c^	7.60 ± 2.60^b^	11.81 ± 2.28^a^
CRE (μmoI/L)	127.76 ± 48.25	157.14 ± 88.35	112.82 ± 61.97
TCHO (mmol/L)	4.00 ± 0.59^b^	6.01 ± 1.76^a^	4.93 ± 1.17^ab^
TG (mmol/L)	1.27 ± 0.27^b^	2.40 ± 1.37^a^	1.24 ± 0.30^b^
VLDL (mmol/L)	0.57 ± 0.12^b^	1.10 ± 0.66^a^	0.56 ± 0.13^b^
WBC (×10^9/L)	6.26 ± 2.35^b^	6.52 ± 2.05^b^	9.57 ± 1.94^a^
LYMPH%	30.53 ± 9.67	35.29 ± 10.52	27.77 ± 2.29
Neutrophil%	64.62 ± 8.59^ab^	56.99 ± 9.29^b^	68.56 ± 1.60^a^
Monocytes	4.80 ± 3.63	4.72 ± 3.11	4.24 ± 1.54

Data represent the means ± SD. Different superscripts indicate significant difference (*p* < 0.05); ALP, alkaline phosphatase; BUN, blood urea nitrogen; CRE, creatinine; TCHO, total cholesterol; TG, triglyceride; VLDL, very low-density lipoprotein; WBC, white blood cell.

### NMR Results

#### ^1^H NMR spectra of serum samples

**Figure 1:**
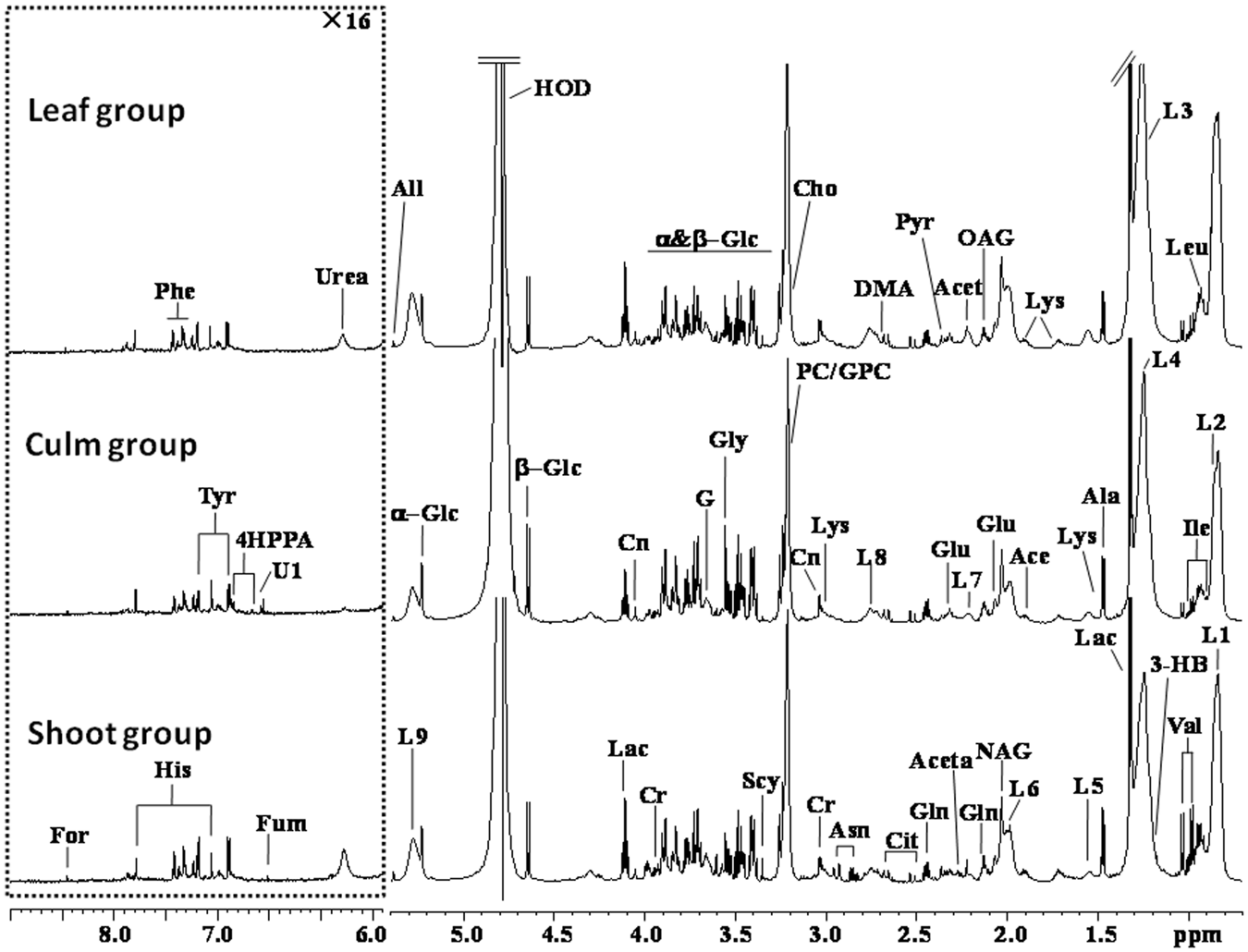
Representative CPMG^1^H NMR spectra (δ0.7–5.4 and δ5.4-9.0) of serum obtained from “leaf” group, “culm” group and “shoot” group. The region of δ5.4–9.0 (in the dashed box) was magnified 16 times compared with corresponding region of δ0.7–5.4 for the purpose of clarity. Keys: 3-HB: 3-Hydroxybutyrate; 4HPPA, 4-Hydroxyphenylpyruvate; Ace: Acetate; Acet: Acetone; Aceta: Acetoacetate; Ala: Alanine; Asn: Asparagine; Cho: Choline; Cit: Citrate; Cr: Creatine; DMA: Dimethylamine; For: Formate; Fum: Fumarate; G: Glycerol; Glc: Glucose; Gln: Glutamine; Glu: Glutamate; Gly: Clycine; GPC: Glycerolphosphocholine; Ile: Isoleucine; L1: LDL, CH_3_-(CH_2_)_n_-; L2: VLDL, CH_3_-(CH_2_)_n_-; L3: LDL, CH_3_-(CH_2_)_n_-; L4: VLDL, CH_3_-(CH_2_)_n_-; L5: VLDL, -CH_2_-CH_2_-C=O; L6: Lipid, -CH_2_-CH=CH-; L7: Lipid, -CH_2_-C=O; L8: Lipid,=CH-CH_2_-CH=; L9: Lipid, -CH=CH-; Lac: Lactate; Leu: Leucine; Lys: Lysine; *m*-I: *myo*-Inositol; NAG: N-acetyl glycoprotein signals; OAG: O-acetyl glycoprotein signals; PC: Phophocholine; Phe: Phenylalanine; Pry: Pyruvate; Scy: Scyllitol; Tyr: Tyrosine; U1: Unknown1; Val: Valine.

Figure 1 shows the representative ^1^H NMR spectra of serum samples obtained from the three different groups and a total of 35 discrepant metabolites were shown in Table 3.

**Table 3:** OPLS-DA coefficients derived from the NMR data of metabolites in serum obtained from different groups.

Metabolites	Correlation coefficient^a^		
	Culm vs Shoot^a^	Leaf vs Shoot^a^	Leaf vs Culm^a^
Valine	−0.865	−0.792	—
Leucine	−0.761	—	—
Isoleucine	−0.793	—	—
Histidine	0.735	—	−0.803
Lysine	−0.699	−0.673	—
Tyrosine	−0.667	−0.726	—
Glycine	0.882	—	−0.938
Alanine	0.635	−0.898	−0.853
Phenylalanine	−0.794	−0.793	−0.749
Dimethylamine	−0.851	—	—
Asparagine	−0.777	−0.795	—
4-hydroxyphenylpyruvate	0.907	—	−0.884
Glutamine	0.742	−0.806	−0.874
Creatine	−0.847	−0.649	0.696
Choline	—	—	−0.686
Fumarate	0.892	—	−0.801
Pyruvate	−0.672	−0.889	—
Citrate	—	−0.802	—
Lactate	−0.639	−0.740	—
Acetone	−0.853	—	0.784
Acetoacetate	−0.819	−0.705	—
Glycerol	—	—	−0.869
Scyllitol (scyllo-inositol)	−0.893	−0.896	—
O-acetyl-glycoprotein	—	−0.634	—
N-acetyl-glycoprotein	—	—	0.863
L1: lipid, ch_3_-(ch_2_)_n_-(ldl)	—	—	0.753
L2: lipid, ch_3_-(ch_2_)_n_-(vldl)	—	0.712	0.726
L3: lipid, ch_3_-(ch_2_)_n_-(ldl)	0.690	0.758	0.724
L4: vldl, ch_3_-(ch_2_)_n_-	0.690	0.755	0.734
L5: vldl, -ch_2_-ch_2_-c=o	—	0.821	0.791
L6: lipid, -ch_2_-ch=ch-	—	0.875	0.965
L7: lipid, -ch_2_-c=o	—	0.856	0.823
L8: lipid, =ch-ch_2_-ch=	—	0.738	0.765
L9: lipid, -ch=ch-	—	0.911	0.963
Allantoin	−0.779	−0.892	—

^a^Correlation coefficients, positive and negative signs indicate positive and negative correlation in the concentrations, respectively. The correlation coefficient of│r│ > 0.632 was used as the cutoff value for the statistical significance based on the discrimination significance at the level of *P* = 0.05 and *df* (degree of freedom) = 8. “—” means the correlation coefficient│r│ is less than 0.632.

#### Multivariate data analysis of NMR data

**Figure 2:**
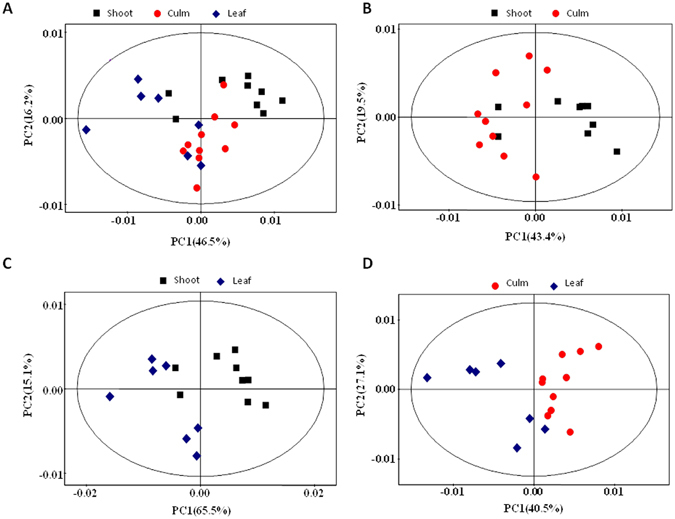
PCA score plots based on the ^1^H NMR spectra of serum metabolites obtained from the “shoot”, “culm” and “leaf” group. (**A**) Total PCA score plots (R^2^X = 62.7%, Q^2^ = 0.479); (**B**) Shoot-Culm (R^2^X = 62.9%, Q^2^ = 0.426); (**C**) Shoot-Leaf (R^2^X = 80.6%, Q^2^ = 0.711); (**D**) Culm-Leaf (R^2^X = 67.6%, Q^2^ = 0.438). Two serum samples from “leaf” group were excluded because it positioned outside the Hotelling’s T^2^ ellipse on the PCA score plot.

A principal component analysis (PCA) was applied to the serum spectral data to investigate the correlations between the three groups. The PCA results showed a tendency to separate the three groups in the score plot (Fig. 2). PLS-DA was conducted on the serum spectra of the “culm”, “shoot” and “leaf” groups.

**Figure 3:**
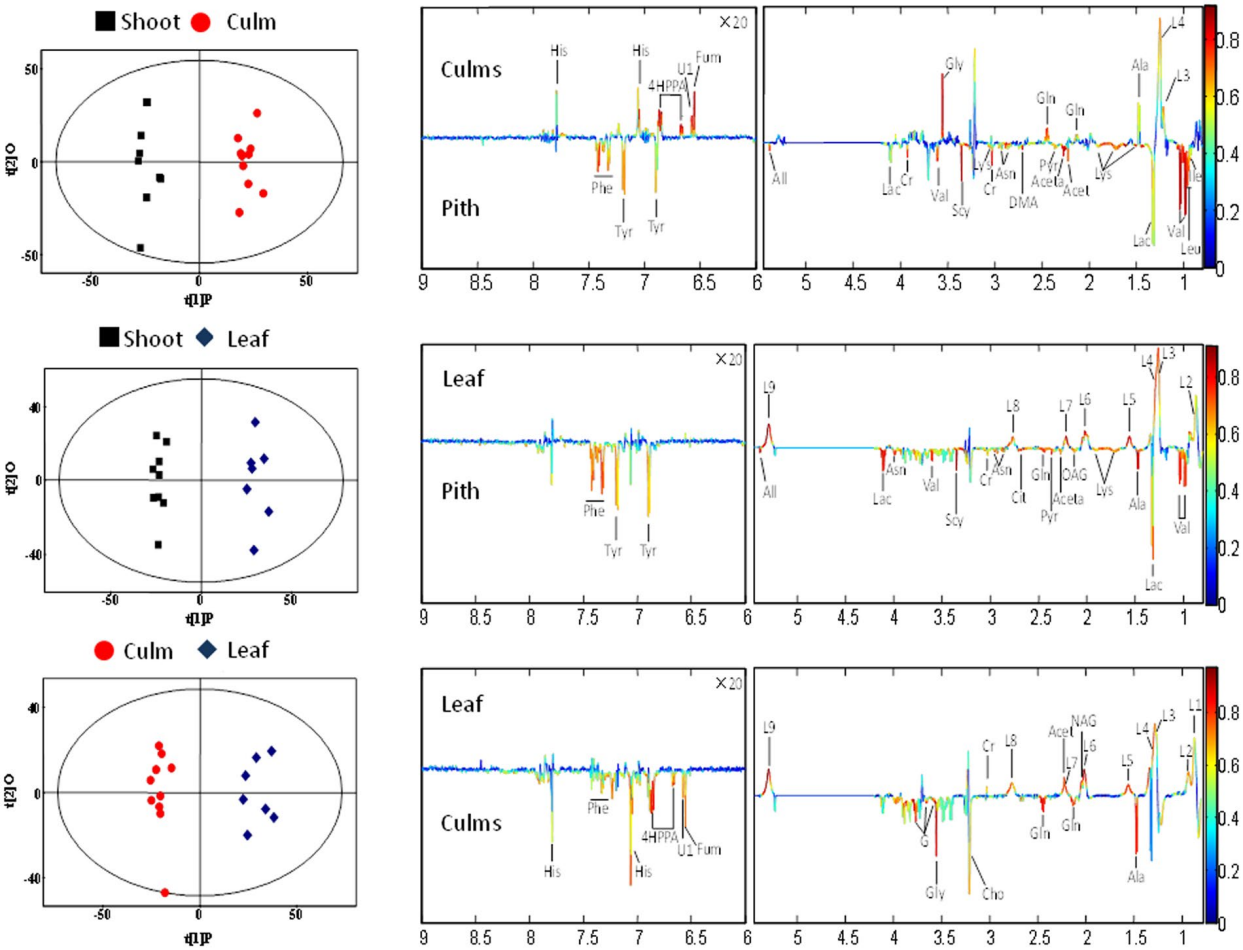
OPLS-DA scores plots (left panel) derived from ^1^H NMR spectra of serum and corresponding coefficient loading plots (right panel) obtained from different groups. The color map shows the significance of metabolites variations between the two classes. Peaks in the positive direction indicate metabolites that are more abundant in the groups in the positive direction of first principal component. Consequently, metabolites that are more abundant in the groups in the negative direction of first primary component are presented as peaks in the negative direction.

Furthermore, the metabolic changes were evident from the separation between the three bamboo forage groups plotted in the PLS-DA when expanding to the supervised multivariable statistical method, OPLS-DA (Fig. 3). The S-plots of OPLS-DA indicated that the metabolic profile of the groups deviated from each other, suggesting that significant biochemical changes were induced by the different bamboo diets. The corresponding coefficient analysis showed that the level of Valine, Leucine, Isoleucine, Lysine, Dimethylamine, Asparagine, Phenylalanine, Tyrosine, Creatine, Pyruvate, Lactate, Acetone, Acetoacetate, Scyllitol and Allantoin in the serum of shoot-fed pandas increased compared with the “culm” group (*p* < 0.05), while the level of Histidine, 4-hydroxyphenylpyruvate, Glutamine, Glycine, Alanine, Fumarate and lipids in the serum decreased (*p* < 0.05, Fig. 3 and Table 3). Compared with the “leaf” group, the level of Valine, Lysine, Asparagine, Phenylalanine, Tyrosine, Glutamine, Alanine, Creatine, Pyruvate, Citrate, Lactate, Acetoacetate, Scyllitol, O-acetyl-glycoprotein and Allantoin in the serum of the “shoot” group increased (*p* < 0.05), and most of the lipid compounds in the serum decreased (*p* < 0.05, Fig. 3 and Table 3). Compared with the “leaf” group, the level of Histidine, Phenylalanine, 4-hydroxyphenylpyruvate, Glutamine, Glycine, Alanine, Choline, Fumarate and Glycerol in the serum of the “culm” group increased (*p* < 0.05), and the level of Creatine, Acetone, N-acetyl-glycoprotein and most of the lipids compounds in the serum decreased (*p* < 0.05, Fig. 3 and Table 3).

### GC-MS Results

**Figure 4:**
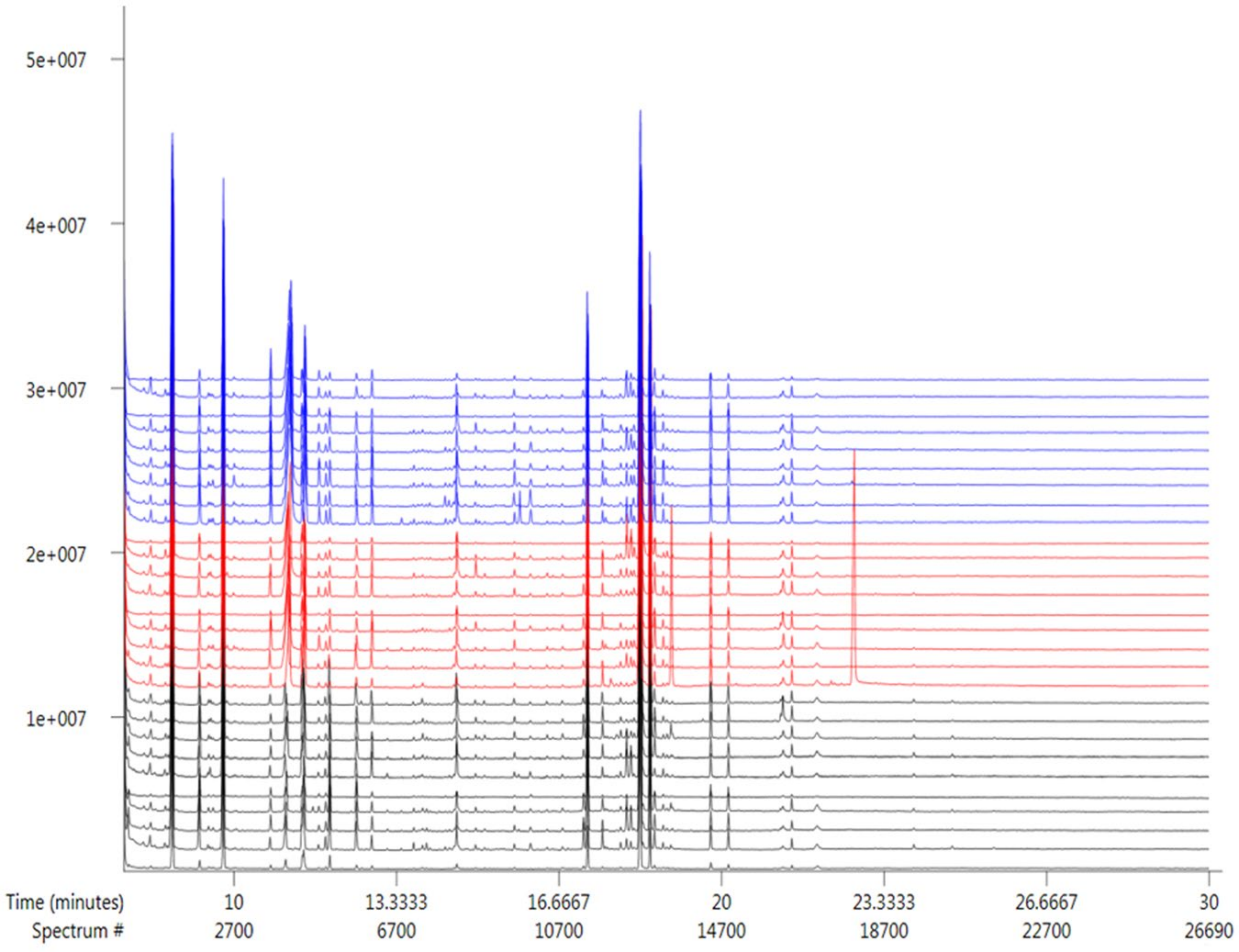
Representative GC-MS total ion chromatograms from the serum samples of “culm”, “leaf” and “shoot” group. Blue: Shoot group; Red: Leaf group; Black: Culm group.

The GC-MS total ion current chromatograms of the 28 serum samples from “culm”, “leaf” and “shoot” group are shown in Fig. 4. There are obvious changes in the three groups. Based on the LECO-Fiehn Rtx5 database and related references, most of the peaks were identified as metabolites, including the following: amino acids, glucose and fatty acids. These metabolites were mainly involved in glucose metabolism, lipid metabolism and amino acid metabolism.

### Multivariate statistical analysis of metabolites

**Figure 5:**
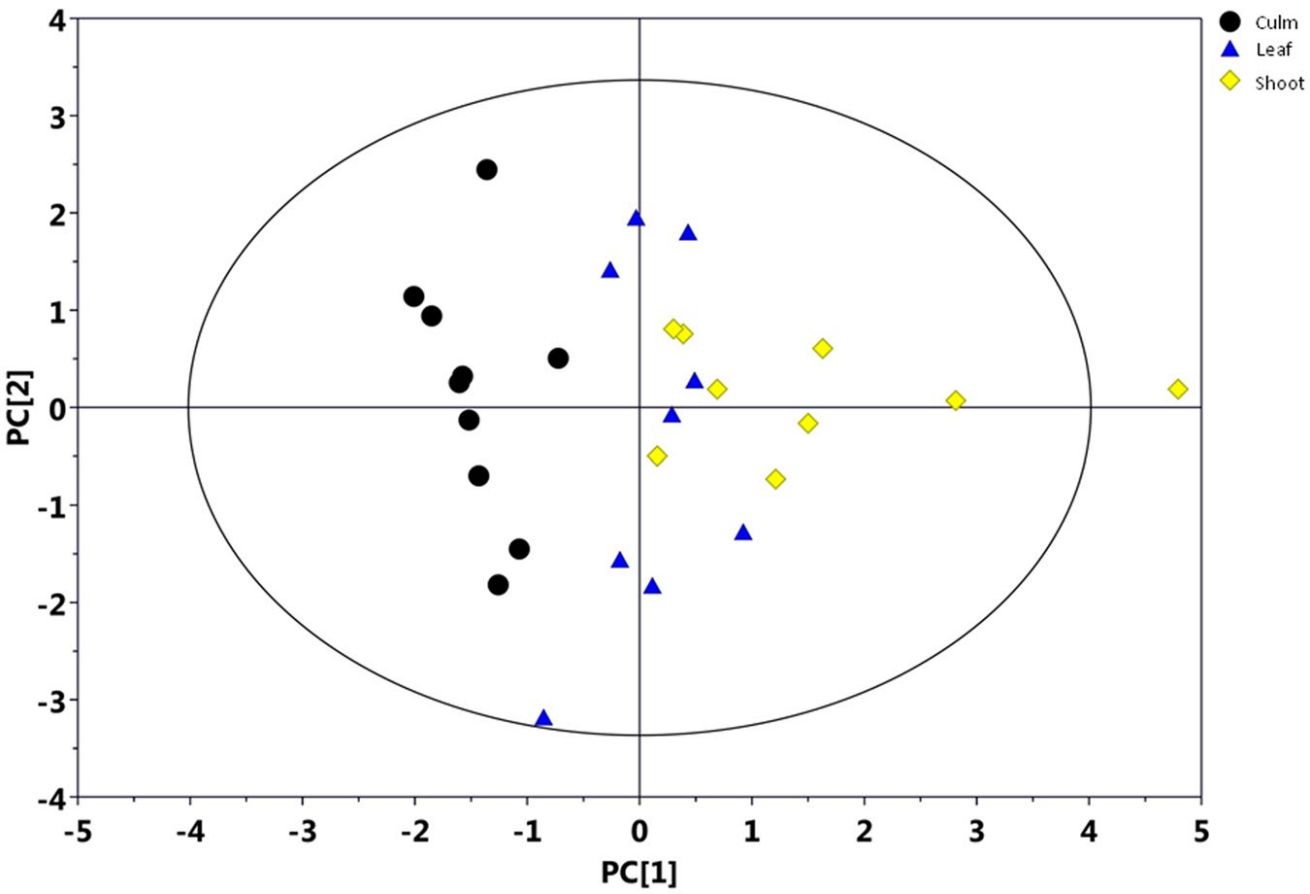
Score plot of PCA model of TOTAL samples.

**Figure 6:**
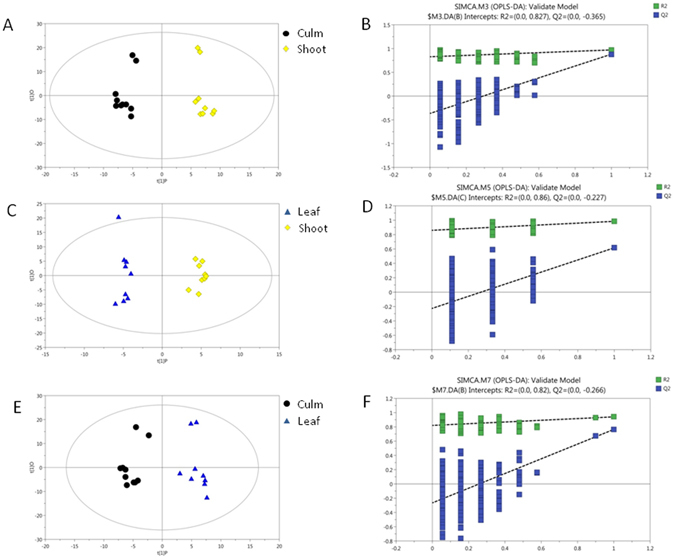
Score plot of OPLS model obtained from Culm vs Shoot (**A**); Leaf vs Shoot (**C**); Culm vs Leaf (**E**).Permutation analysis of OPLS-DA models from Culm vs Shoot (**B**); Leaf vs Shoot (**D**); Culm vs Leaf (**F**). Two hundred permutations were performed, and the resulting R^2^ and Q^2^ values were plotted. (Green triangle): R^2^; (blue square): Q^2^. The green line represents the regression line for R^2^ and the blue line for Q^2^.

Missing values of raw data were filled up by half of the minimum value, which means the peak area of all of samples. There are 107 missing values, and then, 438 peaks were detected and 186 metabolites could be left through an interquartile range denoising method. In addition, an internal standard normalization method used D-Phenylalanine, 2,4-dichloro-N-[(1,1-dimethylethoxy)carbonyl] as the interior label was employed in this data analysis. The resulting three-dimensional data set includes involving the peak number, sample name, and normalized peak area and was analyzed using the SIMCA software package (V14, Umetrics AB, Umea, Sweden) for principal component analysis (PCA) and the orthogonal projections to latent structures-discriminant analysis (OPLS). The PCA showed the distribution of the original unsupervised data structure (Fig. 5). In order to obtain a higher level of group separation and a better understanding of the variables responsible for group classification, a supervised partial least squares discriminant analysis (OPLS-DA)was applied. A 7-fold cross validation was used to estimate the optimal component number of the model and avoid the over-fitting. The R^2^ and Q^2^ intercept values were R^2^ = 0.827, Q^2^ = −0.365 (Culm vs Shoot); R^2^ = 0.860, Q^2^ = −0.227 (Leaf vs Shoot); R^2^ = 0.820, Q^2^ = −0.266 (Leaf vs Culm) after 200 permutations. The low values of the Q^2^ intercept indicate the robustness of the models, and thus show a low risk of over fitting and reliability. Based on the OPLS, a loading plot was constructed, which show the contribution of variables to the differences between each pair of groups (Fig. 6). The OPLS loadings also show the most important variables which were situated fararthest from the origin (Fig. 6). To refine this analysis, the first principal component of the variable importance projection (VIP) was obtained. The VIP values exceeding 1.0 were first identified as changed metabolites. In step 2, the remaining variables, that were different between the two groups, were evaluated for their significance to the model as assessed by Student’s T test (T-test), *P* > 0.05, variables were discarded between two comparison groups. In addition, commercial databases, including KEGG (http://www.genome.jp/kegg/) and NIST (http://www.nist.gov/index.html) were utilized to search for the MS library signals and associated gene expression of metabolites. Table 4 shows the identified differences metabolites in the three groups. Twenty-three metabolites with VIP-values greater than 1.0 and *P* values less than 0.05 were to be significantly different in the different groups. Compared with the “shoot”group, the level of Beta-alanine, Glycine, Alanine, Aminomalonic acid, Glucose, Aconitic acid, 1,5-anhydroglucitol, Threonic acid, Dl-anabasine, Threitol and Arbutin of the culm-fed pandas increased, and the level of Tryptophan, Lysine, Phenylalanine, Fructose, Mannose, Pyruvate, Lactate, D-glyceric acid and D-(glycerol 1-phosphate) decreased. Compared with the “shoot” group, the level of L-cysteine, 1,5-anhydroglucitol, Threonic acid, Dl-anabasine, and Threitol of the “leaf” group increased, and the level of Alanine and Lactate decreased. Compared with the “culm” group, the level of L-cysteine, Mannose, D-glyceric acid and Elaidic acid of the “leaf” group increased, and the level of Serine, Glycine, Alanine, Aminomalonic acid, Glucose, Aconitic acid, Lactate, and Arbutin decreased.

**Table 4:** The fold change in metabolite abundance among the Culm, Leaf and Shoot groups^a^.

Metabolites	r^a^		
	Culm-Shoot	Leaf-Shoot	Leaf-Culm
Tryptophan	6.46E − 01		
Lysine	7.10E − 01		
Serine			6.01E − 01
Beta-alanine	2.81E + 00		
Phenylalanine	6.15E − 01		
L-cysteine		2.07E + 00	2.16E + 00
Glycine	1.97E + 00		4.41E − 01
Alanine	1.37E + 00	7.00E − 01	5.10E − 01
Aminomalonic acid	2.18E + 00		5.89E − 01
Fructose	2.89E − 01		
Mannose	3.41E − 01		2.49E + 00
Pyruvate	6.27E − 01		
Glucose	8.65E + 01		1.59E − 01
Aconitic acid	4.83E + 00		2.74E − 01
1,5-anhydroglucitol	1.42E + 00	1.52E + 00	
Lactate	7.38E − 01	6.17E − 01	1.33E − 01
D-glyceric acid	5.98E − 01		1.84E + 00
D-(glycerol 1-phosphate)	7.09E − 01		
Threonic acid	1.98E + 00	1.94E + 00	
Dl-anabasine	5.58E + 00	3.08E + 00	
Threitol	1.55E + 00	2.18E + 00	
Elaidic acid			1.83E + 00
Arbutin	2.58E + 00		4.44E − 01

^a^All of the metabolites’ similarity >700, fold change >2 or <0.5. The numbers mean the fold change of the former group to the latter group. If the number <1, it means that the amount of the metabolite in the former group is lower than in the latter group; and if the number >1, it means that the amount of metabolite in the former group is higher than in the latter group.

## Discussion

Bamboo is the staple food of giant pandas, however, there are various parts of bamboo for giant pandas to consume. Previous studies on captive giant panda nutrition have not explored the effect of different single- plant bamboo parts on the metabolism and health of pandas^19^. The result of the digestibility trial shows that the CP digestibility is higher in “shoot” group than “leaf” group (*p* < 0.05), and “leaf” group is higher than “culm” group (*p* < 0.05). Interestingly, the CF composition is much higher in “culm” group than in “leaf” group (*p* < 0.05), and “leaf” group is higher than in “shoot” group (*p* < 0.05) (Table 5). Based on these results, we speculated that high level of fiber will reduce the efficiency of the CP digestibility, which is similar in other monogastric mammals^20, 21^. Therefore, although the amount of daily CP intake in “shoot” group was similar to that of the “leaf” group, the digestible CP in “shoot” group was much higher than “leaf” group (*p* < 0.05).

**Table 5:** The nutrient composition of the culm in *Phyllostachys B*., leaf in *Bashania F*. and shoot in *Qiongzhuea O*.

Nutrients	Bamboo Species and plant parts		
	*Phyllostachys bisseti*	*Bashania fargesii*	*Qiongzhuea opienensis*
	Culm	Leaf	Shoot
CP%	1.76 ± 0.27^b^	15.68 ± 1.47^a^	31.10 ± 1.54^c^
EE%	1.65 ± 0.47^b^	3.31 ± 0.76^a^	3.39 ± 0.84^a^
CF%	44.38 ± 3.56^b^	22.12 ± 2.48^c^	13.25 ± 0.95^a^
Energy (MJ/Kg)	18.50 ± 0.07	18.14 ± 0.34	16.64 ± 0.33

The daily EE intake from leaves was the highest, while the digestibility of EE from leaves was the lowest. In contrast, the daily EE intake from culms was the lowest, while the digestibility of EE from culms was the highest. This may be related to the low enzymatic activity of the lipase in the intestinal tract of giant pandas^22^. The EE in the leaves was too high for giant pandas to fully digest, which might result in the lower digestibility of EE from the leaves than that from the culms. These results show that the nutrients of bamboo will affect their gastrointestinal digestion and absorption capacity.

Maintenance of a stable body mass is often used as an indicator of health. Our data showed there was a significant difference in mass gain that correlated with experimental diet groups. During our study “shoot” group had higher body mass gain than “culm” and “leaf” groups. The “culm” pandas digested and absorbed the most calories and fiber compared to pandas in the other groups, and leaf group pandas digested and absorbed more calories than “shoot” pandas, however, “culm” and “leaf” pandas digested and absorbed less protein than shoot pandas. Therefore, we speculated that fiber digestion demands energy, and demands more energy to consume than it gives back in caloric value. So it takes physical effort, and energy to eat fiber, but no calories are returned and is thus negative in net caloric value. To explore the accuracy of this assumption, metabonomic methods were used to analyze the serum metabolites of the different treatment-fed pandas.

Creatine is involved in major energy metabolism in vertebrates^23, 24^, and contains more energy than ATP. High levels of 1,5-anhydroglucitol content in serum is an indicator of hypoglycemia^25, 26^. “culm” and “leaf” groups had lower creatine contents and higher 1,5-anhydroglucitol levels in serum than the “shoot” group, which shows that the energy reserve in the “culm” and “leaf” groups is lower than the “shoot” group pandas. Moreover, the “leaf” group had lower tricarboxylic acid cycle activity than “shoot” group, with lower citrate concentrations, because pyruvate is an important substrate of tricarboxylic acid cycle and citrate is involved in tricarboxylic acid cycle^27^. The “culm” group had a more compromised energy source and tricarboxylic acid cycle activity compared to the “shoot” group with lower concentrations of fructose, pyruvate, mannose, and creatine, and higher levels of aconitic acid, fumarate, 1,5-anhydroglucitol. The “culm” group had higher levels of alanine and aminomalonic acid than the “shoot” group most likely because alanine can react with oxomalonate to create the bi-products aminomalonic acid and pyruvate^28^. In addition, the fat intake was lower in the “culm” group than in the “shoot” group, however, the serum lipid level was higher in the “culm” animals than in the “shoot” group. These data show that “culm” group pandas were in short supply of energy with depressed tricarboxylic acid cycle activity, and mobilized amino acids, glycogen and lipids to compensatively trigger the tricarboxylic acid cycle to meet their energy requirements^29–31^. Thus, culm pandas were in trend of negative energy balance. Usually, negative energy balance is harmful to health^32, 33^. Therefore, we conclude that fiber digestion demands more energy to consume than it gives back in caloric value, which lead to lower growth rate of culm- and leaf-fed pandas.

Some studies show that ruminant and monogastric herbivores can use the energy produced through fiber fermentation and promote the body mass growth^34, 35^. However, the monogastric non-herbivores are different from the ruminant and monogastric herbivores, and a high fiber diet is not beneficial for energy reserves and body mass growth^36, 37^. Therefore, even though bamboo is the staple food of giant pandas, the bioavailability of fiber is relatively low, and high level fiber might reduce the efficiency of the CP digestibility. These results are in accordance with the conclusion of the previous studies which show that giant pandas’ gut microbiota is distinct from those of red pandas and other herbivores, and the coevolution of the giant panda and its gut microbiota are aberrant^38–40^, even though it has been 4.2 million years since they evolved from carnivory to herbivory^41^. Moreover, compared with other groups, shoot ingestion improved the most amino acids concentrations in the blood serum of “shoot” group. Combined with higher average body mass gain in “shoot” group, we also found that more digested and absorbed protein in bamboo enhanced body growth. Overall, because of the digestion, absorption and metabolism characteristics of pandas, different bamboo parts can affect the maintenance of body mass for pandas, thus impacting health.

The “leaf” group pandas had the highest triglyceride and total cholesterol concentrations compared to the other groups, and the “culm” group pandas were in negative energy balance. The “shoot” group had a greater increase in mass, however, the “shoot” group had higher levels of ketone bodies, lactate, urea, BUN, BUN/CRE than “culm” and “leaf” fed pandas, and “shoot” group pandas had higher WBC and neutrophils (Table 2) implying a potential inflammation reaction in the body. Because “shoot” fed pandas ingested more protein and amino acids than the other experimental groups, they had higher BUN and BUN/CRE, and metabolic burden aggravates the production of ketone bodies and lactate^42–44^. If pandas consume “shoot” for a long time, they may potentially suffer from nephropathy or other diseases. Because the high levels of BUN and BUN/CRE are the indicators of low glomerular filtration rate and kidney damage. Continuous high level of BUN tends to cause kidney disease. In addition, according to our husbandry observations, the number of female pandas in oestrous will decrease when culms are lacking in the diet (unpublished data). This phenomenon might be the result of lower fiber intake, because culms contain higher fiber than the other bamboo parts tested. In summary, different bamboo parts can affect the health of pandas.

The results of this study are meaningful not only for the management of *ex-situ* giant pandas but also for the conservation of the *in-situ* population as well. Bamboo species consumed by giant pandas vary with different habitat and elevations. In the Qinling Mountains nine bamboo species belonging to five genera have been described, however giant pandas prefer to consume *Bashania fargesii* and *Fargesia spathacea* ^4, 45^. These two species are the dominant native bamboos in the region. Schaller (1985) observed that giant pandas in the Wolong Nature Reserve fed mainly on *Sinarundinaria fangiana* and *Fargesia spathacea*, while *Sinarundinaria nitida* was also consumed, but to a much lesser extent^3^. Older studies reported that giant pandas in the Min Mountains consumed a single bamboo species, whereas animals in the Liang Mountains forage upon five bamboo species^46^. These data show that the nutrition intake of the wild pandas in the different reserves might vary significantly, this may affect wild giant panda health and reproductive abilities. As a result, it is necessary to estimate whether bamboo species found in the various nature reserves could provide a balanced nutrition for wild giant pandas, and in addition aid conservation managers in selecting the appropriate habitat for giant panda reintroductions.

## Conclusion

The “culm” group absorbed the highest amount of calories and fiber, but was in short energy supply with a depressed tricarboxylic acid cycle activity, therefore, digestion of fiber requires energy input and yields low caloric extraction from the culm and leaf. In addition, the high level fiber will reduce the CP digestibility for giant panda. So, the high level of fiber not only make pandas in trend of negative energy balance, but also disturb the digestion of CP, these characteristic is similar with monogastric non-herbivores. The “culm” and “leaf” groups absorbed less amount of protein, and had a lower body mass increase than the “shoot” group, this showed that protein was benefit for body mass gain. However, long-term intake of shoots may have a potentially negative effect on the health. Therefore, the balance of an annual plant parts diverse bamboo diet is important for the health of giant pandas. In addition, we need to protect and conserve the biological diversity of bamboo in giant panda habitat, which is crucial for the survival and health of the *in-situ* population.

## Methods

### Study subjects and husbandry methods

A total of 17 adult captive giant pandas from the Chengdu Research Base of Giant Panda Breeding were the subjects of this study. There were three food groups based on bamboo species (Table 6). None of the female pandas were in estrus, pregnant, or nursing during the study. The bamboo species provided were *Phyllostachys bissetii* (culm group) from which the pandas only consume the culm, *Bashania fargesii* (leaf group) in which the pandas only consume the leaves and bamboo shoots of *Qiongzhuea opienenss* in which the pandas only consume the shoot (shoot group). Following the normal feeding protocol of the Chengdu Research Base of Giant Panda Breeding, all of the pandas were fed bamboo *ad libitum*, with experimental treatments lasting for 20 consecutive days. The supplemented diets were provided at the same mass every day among the three groups. The daily feed intake was recorded. The giant pandas were weighed at the beginning of each dietary treatment and every 15 days after consecutively consuming one specific bamboo species.

**Table 6:** Information of the adult captive giant pandas.

Bamboo Species	Plant parts intake	Sex		Age	Date	Supplemented diets
		Male	Female			
*Phyllostachys B*.	Culms	4	6	8–17	Mar-Apr 2014/2015	Panda Cake; Apple
*Bashaniafargesii F*.	Leaves	4	5	7–16	Nov-Feb 2014/2015	Panda Cake; Apple
*Qiongzhuea O*.	Shoots	4	5	8–16	May-Jun 2014/2015	Panda Cake; Apple

### Blood collection

Twenty-eight blood samples in total were collected from three groups after single bamboo plant parts intake for 20 consecutive days: nine blood samples from the “shoot” group, ten from the “culm” group and nine from the “leaf” group. Blood samples were collected from the giant pandas during routine physical examinations by experienced staff veterinarians at the Chengdu Research Base of Giant Panda Breeding. All sample collection and utility protocols in this study were approved by Chengdu Research Base of Giant Panda Breeding. The experimental procedures were fully in compliance with the current laws on animal welfare and research in China.

### Blood chemistry measurements

Chemistry analyses were performed with an automatic biochemical analyzer (OLYMPUS AU-2700), which included alkaline phosphatase (ALP), blood urea nitrogen (BUN), creatinine (CRE), total cholesterol (TCHO), triglyceride (TG), very low-density lipoprotein (VLDL), white blood cell (WBC), LYMPH, Neutrophil and Monocytes.

### Apparent digestibility trial

The apparent digestibility trial was conducted for consecutive 3 days after one-plant bamboo part intake for 20 days. All of the food provided, orts and feces were weighed. To calculate the amount of bamboo intake, three control groups were made to test the water loss under the same environmental conditions as the study animals were kept in. Samples of bamboo which the pandas ate and feces were collected twice per day, weighed and stored for nutrient composition analysis. The items of the nutrient composition include dry matter (DM), crude protein (CP), ether extract (EE), crude fiber (CF) and energy.

Dry Matter was measured using an oven drying method (DHG-9070A), crude protein was measured by kjeldahl method (K1100, Automatic kjeldahl apparatus), ether extract was measured by Soxhlet extraction method (SOX500), crude fiber was measured by Acid base digestion method (ANKOM A200i), energy concentration in bamboo and feces was measured by oxygen bomb calorimeter calorimetric method (PARR 6400 Calorimeter).

The apparent digestibility macro-nutrients were calculated as follows:

$${\rm{Apparent}}\,{\rm{digestibility}} \% =\frac{{\rm{nutrient}}\,\mathrm{digested}\,(\mathrm{nutrient}\,\mathrm{consumed}\,-\,{\rm{nutrient}}\,{\rm{excreted}})}{{\rm{nutrient}}\,\mathrm{consumed}\,(\mathrm{nutrient}\,\mathrm{offered}\,-\,{\rm{nutrient}}\,{\rm{refused}})}$$

### Sample preparation of ^1^H-NMR metabolomics of serum

serum samples were removed from −80 °C storage and thawed at room temperature. 200 μL of serum and 400 μL of phosphate buffer compounded by D_2_O (Deuterium Oxide) were pipetted individually and mixed together for field-frequency lock. After centrifugation at 4 °C and 12,000 rpm for 10 min, 560 μL of the supernatants were transferred into NMR tubes (Norell, ST500-7) for ^1^H-NMR analysis^47^.

^1^H-NMR spectra of the serum samples were acquired at 298 K on a 600 MHz high resolution spectrometer (Agilent 600DD2, USA), operating at a ^1^H frequency of 600.11 MHz with three high resolution resonance probes. A total of 64 transients were collected with a spectral width of 20 ppm. The 90° pulse length was adjusted to 10 μs and the recycle delay is 2 s. A water-presaturated Carr-Purcell-Meiboom-Gill (CPMG) pulse sequence (RD -90sonτ-180-Pτ)_n_-ACQ) was used to record the NMR spectrum. A total spin-spin relaxation delay(2 nτ) of 200 ms was employed.

### Sample preparation of GC-MS metabolomics

Take 100 μL sample into 1.5 mL EP tubes. Add 0.4 mL methanol and 20 μL of L-2-Chlorophenylalanine (0.1 mg/mL stock in dH_2_O) as an internal standard and vortex for 10 s. Centrifuge for 15 min at 12000 rpm, 4 °C. Transfer the supernatant (0.39 mL) into a fresh 2 ml GC/MS glass vial. Take 13 μL from each sample and pool as QC sample. Dry in a vacuum concentrator without heating. Add 60 μL of methoxyamination reagent (20 mg/mL. in pyridine) and incubate for 20 min at 80 °C. Add 80 μL BSTFA regent (1% TMCS, v/v) to the sample aliquots and incubate for 1 h at 70 °C. Add 10 μL FAMEs (Standard mixture of fatty acid methyl esters, C8-C16:1 mg/mL; C18-C24:0.5 mg/mL in chloroform) to the QC sample after it cools to room temperature. Mix well for GC-MS analysis.

GC-MS analysis. GC-MS analysis was performed using an Agilent 7890 gas chromatograph system coupled with a Pegasus 4D time-of-flight mass spectrometer. The system utilized a DB-5MS capillary column coated with 5% diphenyl cross-linked with 95% dimethylpolysiloxane (30 m × 250 μm inner diameter, 0.25 μm film thickness; J&W Scientific, Folsom, CA, USA). A 1 μL aliquot of the analyte was injected in splitless mode. Helium was used as the carrier gas, the front inlet purge flow was 3 mL min^−1^, and the gas flow rate through the column was 20 mL min^−1^. The initial temperature was kept at 80 °C for 1 min, then raised to 295 °C at a rate of 10 °C min^−1^, then kept for 7.5 min at 295 °C. The injection, transfer line, and ion source temperatures were 280, 280, and 220 °C, respectively. The energy was −70 eV in electron impact mode. The mass spectrometry data were acquired in full-scan mode with the m/z range of 85–600 at a rate of 20 spectra per second after a solvent delay of 465 s.

### Data analysis

The mean daily food intake, nutrient digestibility, daily body mass gain, and conventional serum biochemical parameters were determined statistically relevant by one-way ANOVA analysis with SPSS 17.0 software (SPSS Inc., Chicago, IL). The distribution and variance of the data were tested before ANOVA analysis to ensure the parametric tests could be used. Datasets were performed by using post-hoc tests (LSD) for multiple comparisons to determine the statistical differences between groups. Data is presented as mean ± SD. The level of significance used was *p* < 0.05.

Data of ^1^H-NMR: The ^1^H-NMR spectra were processed using MestReNova software (V7.0, Mestrelab Research, Santiago de Compostella, Spain). All spectra were manually corrected for Fourier transformation, phase adjustment, baseline distortions and calibration. The signal-noise ratio was increased by multiplying an exponential function with a line-broadening factor of 1 Hz before Fourier transformation. The spectra of serum were referenced to the peak of the lactate at a chemical shift of δ1.33 ppm. The serum spectra integrating range and the signal integral computed in 0.002 ppm intervals across the region d 0.7–9.5 ppm. The discarded regions include 5.5–6.0 ppm for urea and 4.450–5.214 ppm for H_2_O.

Multivariate data analysis was performed on the normalized NMR datasets with the software package SIMCA-P+ (V11.0, Umetrics AB, Umea, Sweden). Principal component analysis (PCA) was conducted by using mean center scaling on the dataset to overview the intrinsic similarity/dissimilarity within the dataset.

Projection to latent structure–discriminant analysis (PLS–DA) and orthogonal projection to latent structure–discriminant analysis (OPLS–DA) were further conducted to obtain the metabolites with significant contributions to intergroup differentiation by using unit-variance scaled NMR data as the X-matrix and class information as the Y-matrix^48^. The quality of the model was assessed by the parameters R^2^X and Q^2^, which represent the total explained variations for the X matrix and the model predictability, respectively. The models were certified by using a six-segment cross validation^49, 50^. The metabolites associated with the group separations were indicated by coefficient coded loading plots calculated by back transformation of the loadings. *P* values were calculated by the correlation coefficient, and the correlation coefficient was calculated by the SIMCA software package (V14, Umetrics AB, Umea, Sweden). The correlation coefficient of│r│ > 0.632 was used as the cutoff value for the statistical significance based on the discrimination significance at the level of *P* = 0.05 and df (degree of freedom) = 8.

Data of GC-MS: Chroma TOF4.3X software of LECO Corporation and LECO-Fiehn Rtx5 database were used for raw peaks selection, the data baselines filtering and calibration of the baseline, peak alignment, deconvolution analysis, peak identification and integration of the peak area^51^. The RI (retention time index) method was used in the peak identification, and the RI tolerance was 5000. GC-MS spectral data were imported to SIMCA-P+ for principal components analysis (PCA), and orthogonal partial least-squares discriminant analysis (OPLS-DA).Variable Influence on Projection (VIP) values, which correspond to the importance of the variables were calculated, and variables with a VIP value larger than 1.0 were considered significant and used for further analysis and identification of the responsible peak(s) within the spectrum.
